# The Social Support Network of Adults with an Autism Spectrum Condition: An Exploration Using the Network in Action-Questionnaire

**DOI:** 10.1007/s10803-022-05467-5

**Published:** 2022-02-19

**Authors:** Rinske M. van den Heuvel, Michel Wensing, Hilde M. Geurts, Jan-Pieter Teunisse

**Affiliations:** 1 Leo Kannerhuis, Youz (Parnassia Group), Stationsweg 49, 6861 EE Oosterbeek, The Netherlands; 2 HAN University of Applied Sciences, Nijmegen, The Netherlands; 3 General Practice and Health Services Research, University Hospital Heidelberg, Heidelberg, Germany; 4 Department of Psychology, University of Amsterdam, Amsterdam, The Netherlands

**Keywords:** Adults, Assessment, Autism, Social network, Social support

## Abstract

Actively involving the network during treatment, as recommended in Autism Spectrum Condition (ASC) guidelines, can be facilitated with the Network in Action-Questionnaire (NiA-Q), which identifies the current and potential sources of social support. The aims of this study were to (1) examine the factor structure of the NiA-Q and (2) to explore the self- and proxy-report on the social network. Before the start of treatment in a mental health institution, 193 adults with an ASC diagnosis and 84 proxies completed the NiA-Q. Factor analysis showed two factors: positive social support and interpersonal distress. Self- and proxy-report on the NiA-Q did not differ for most variables, except for social network wishes. The NiA-Q provides a basis for network involvement and strengthening.

Multiple studies show a relationship between one’s social support network and mental well-being in the general population (e.g., Gariépy et al., 2016; Santini et al., 2015). In psychiatric populations, the support of one’s social network plays an important role in recovery from mental health problems (Pernice‐Duca, 2010) and perceived social support predicts later psychiatric symptoms (Rogers et al., 2004). Moreover, collaborating with relatives resulted in improved treatment outcomes in patients with general psychiatric problems (e.g., Lyman et al., 2014). The relationship between social support and mental well-being has also been shown in Autism Spectrum Condition (ASC) samples. For example, perceived social support is positively associated with quality of life (Bishop-Fitzpatrick et al., 2018; Renty & Roeyers, 2006) and being satisfied with current social contacts is positively related to feeling happy (Deserno et al., 2017) in adults with an ASC diagnosis and without intellectual disability (ID). Therefore, it is not surprising that ASC intervention guidelines recommend involving the social network of patients^1^ with an ASC during treatment^2^ (Kan et al., 2013; National Institute for Health and Care Excellence, 2016). To our knowledge, there are no studies that looked specifically at the effects of increasing involvement of network members in adult ASC samples during mental health interventions. To facilitate professionals in the process of involving network members during treatment, a measurement instrument designed for clinical practice could gather practical knowledge for the professional and patient with ASC on how to achieve increased network involvement.

Although a range of instruments aimed at investigating the social support or social network are available, see for an overview for example (Siette et al., 2015), they often have drawbacks for use in clinical practice. For example, they assess perceived social support only in a more general approach and do not provide information by which specific network member this social support is given (e.g., Zimet et al., 1988) or only from specific persons, such as a partner or a nominated friend (Hanssen et al., 2019; Stansfeld & Marmot, 1992). However, even instruments with an assessment of the social support network more relevant for clinical practice (e.g., Tracy & Whittaker, 1990), either do not focus on the social support network related to treatment goals or do not ask explicitly about the patient’s wishes regarding one’s network. Hence, most instruments do not provide concrete clues for increased involvement of network members based on patients’ needs and expectations, as a combination of information with respect to the social support network is required. However, this is precisely what seems most needed for professionals to be able to involve network members in clinical care: an easy application of knowledge gathered by the instrument to take action regarding the social support network. Therefore, to increase involvement of network members of patients with an ASC in mental health care, we present the results of the first clinical application of the Network in Action-Questionnaire (NiA-Q). The NiA-Q is a new instrument for clinical practice that gives insight into the actual and potential social support of a patient and into patient’s needs and expectations regarding his or her social network via self- and proxy-report.

The NiA-Q takes the personal goals of a patient as starting point and then investigates which network members play a supporting or hindering role regarding these goals. As social support can be conceptualized along different dimensions (Chronister et al., 2006), the instrument focuses both on structural components of social support, such as size of the network and frequency of contacts, and functional dimensions, such as perceived social support and interpersonal distress. Functional aspects of social support are also measured in a more detailed approach, aiming to capture three functional components of social support: *Emotional support*, *Practical support* and *Interpersonal distress*. With this latter factor, also negative interactions and influences of network members are taken into account. These three functional components are associated with mental health and well-being (e.g., Beutel et al., 2017; Smith et al., 2012) and provide the clinician with detailed information on the kind of social support that the patient receives from which network member. Due to the specification of support per treatment goal, the professional and patient get insight for which goals or life areas support might be lacking or for which someone might perceive hindering. This differentiates the NiA-Q from other existing instruments and makes it more beneficial and practical for clinical use. For example, if the NiA-Q indicates that an individual perceives very little social support for a certain treatment goal and there is a relevant network member that is not yet involved, the professional and patient can try to involve this person more. Additionally, satisfaction with the current network and what an individual wants to change in the network to increase satisfaction, the “social network wishes”, are assessed. In other words, the NiA-Q can serve as a measurement tool to systematically examine possibilities for involving and strengthening the social support network.

However, besides a clinical use, the data collected via the NiA-Q can provide further scientific knowledge on the characteristics of the social support network in patients with an ASC. The research that has been conducted on social support in adults with an ASC shows for example that people with an ASC and without ID have a smaller social network and are on average less content with their social network than nonautistic individuals (van Asselt-Goverts et al., 2015). Adults with an ASC and without ID often feel isolated and are longing for more emotional intimacy with others (Müller et al., 2008). Also, people with an ASC and without ID experience in general low levels of social support compared to nonautistic adults and adults with attention deficit/hyperactivity disorder (ADHD) (Alvarez‐Fernandez et al., 2017). This lower score specifically concerned perceived social support provided by friends, and not by family members and significant others. Besides information on the general characteristics on the social support network, the NiA-Q can be used to investigate changes in the social support network during the course of treatment. So, the NiA-Q serves both clinical and scientific purposes.

Clinicians can more adequately capitalize on the social network if they know on which domains of the social support network patients and proxies (e.g., partner, parent, or close friend) have overlapping or diverging views. For example, if there are domains on which the perspective of patients and proxies systematically differ, these domains might need additional attention. Likewise, if research shows that there are no substantial differences between these perspectives, this might lower the need for an additional proxy-report in adults with an ASC. Although self- and proxy-report on social networks appeared to overlap substantively in people with mental health problems, there are also important discrepancies (Pescosolido & Wright, 2004). For example, their perceptions on the availability of persons at the center of their network differed: the proxies reported parents, children and siblings as more available for support than the self-report demonstrated. Another study in individuals with mental health problems found strong agreement between self- and proxy-report regarding factual information about network members (e.g., frequency of face-to-face contact), but the two perspectives showed lower correlations on ratings of relationship quality, indicating less agreement in perspectives (Stein et al., 1995).

However, for adults with an ASC, there is little knowledge on the agreement between self-report and proxy-report (Sandercock et al., 2020). In a recent study, the agreement between self- and proxy-report in adults with an ASC and without ID appeared to differ per domain (Sandercock et al., 2020). For example, proxies reported significantly fewer daily living skills and lower quality of life than adults reported themselves. Also, proxies reported that more additional services were needed than adults reported themselves. By contrast, no differences between the two forms of report were found regarding current ASC symptoms (Sandercock et al., 2020), although this finding is inconsistent with previous research (Lever & Geurts, 2018). However, it is unknown whether there are informant discrepancies when it comes to the social support network of adults with an ASC, even though information about possible discrepancies could be of value for effective support. Therefore, we also explored a version of the NiA-Q which is completed by someone close to the patient.

In this study, the results of the first application of the NiA-Q in clinical practice are presented. The study had multiple aims. Firstly, we examined the items on social support which are part of the NiA-Q, to investigate whether the three factors *Emotional support*, *Practical support*, and *Interpersonal distress* were found within our data. Secondly, the characteristics of the social support network of patients with an ASC as reported by patients themselves and by a close network member were compared. Thirdly, we investigated and compared the wishes regarding the social network of patients, as reported by patients and their proxies.

## Methods

### Participants

All participants and their proxies were recruited via a mental health institution specialized in treatment of individuals with autism and without ID in the Netherlands. This mental health institution provides a highly specialized form of clinical care and is situated across four different locations throughout the country with both inpatient and outpatient facilities. The patient population is characterized by having co-occurring conditions or problems besides a diagnosis of an ASC (e.g., depressive disorder, anxiety disorder or problems within the family system) and they have often received previous treatment at other mental health institutions that had been unsuccessful. Data collection took place in 2015 and 2016. Inclusion criteria for the adults with an ASC were (1) diagnosed with autism, Asperger’s syndrome or PDD-NOS by a psychiatrist or psychologist based on DSM-IV (American Psychiatric Association, 2000) or with autism spectrum disorder based on DSM-5 (American Psychiatric Association, 2013); (2) being a patient of the specialized autism center; and (3) age 18 years or older. All participating adults were instructed to ask at least one proxy to fill out the proxy-version of the NiA-Q. This proxy was an adult chosen by the patient, whom the patient considered as an important and close network member. Apart from that, there were no specific inclusion criteria for the proxy.

### Procedure

The study was approved by the Medical Ethics Committee of Amsterdam University Medical Center, classifying as not falling under the Dutch Act on Medical Research Involving Human Subjects (WMO) (reference number W15_265 # 15.0312). Participants were recruited after registering for treatment at the mental health institution. After giving their informed consent, participants completed the questionnaires online. All participants were asked if a close other would fill out the NiA-Q proxy-version. If they and the proxy agreed, the proxy completed this questionnaire online. After the patient and proxy had completed the NiA-Q, the therapist discussed the relevant outcomes and possible actions regarding the network with them. However, in this study, we focus only on the data of the NiA-Q and not on follow-up actions.

### Measures

#### SRS-A

The SRS-A (Constantino, 2002) is the adult version of the SRS (Constantino & Gruber, 2005; Dutch version: Noens et al., 2012). It measures autistic traits in 65 items, which are answered on a 4-point Likert scale (i.e., ‘not true’, ‘sometimes true’, ‘often true’, and ‘almost always true’). The instrument consists of the subscales social awareness, social communication, social motivation, and autistic mannerisms, and a total score. Higher scores indicate more autism traits. The validity and reliability of the Dutch version of the SRS-A showed to be adequate (e.g., α = 0.95) and the cut-off score is 54 (Noens et al., 2012). In the current study, the SRS-A was used for descriptive purposes.

#### NiA-Q

##### Development of Instrument

The development process of the instrument existed of two phases (see also Osinga et al., 2016). In the first phase, the wishes and needs of professionals regarding the instrument were collected in qualitative interviews. In total, 22 professionals involved in the inpatient or outpatient treatment of patients with autism (e.g., psychiatrists, social workers, therapists, social psychiatric nurses) were interviewed. Professionals were asked which aspects of the social network of patients they considered as important for (1) the complexity of the psychological problems of a patient, (2) selecting actions to involve the social network of a patient in treatment, and (3) finding possibilities for strengthening the social network of a patient during treatment. Additionally, professionals gave their opinion on the design and format of the instrument. Interviews were verbatim transcribed and analyzed using Atlas.ti 7.0 according to the general inductive-approach (Thomas, 2006). The result of this phase was a first version of the NiA-Q, which was based on both the relevant literature on social networks and important factors of the social network that emerged from the interviews.

In the second phase, the first version of the instrument was pilot tested by 12 professionals, 13 patients with an ASC and 22 proxies. Professionals evaluated the questionnaire with respect to the overall impression of the instrument, its quality, its use, and the integration of it within usual care. Patients and proxies gave their evaluation on their experiences with the digital questionnaire, on what questions were lacking in the instrument, on their experiences with discussing the results of the questionnaire with a therapist and possible improvements of the questionnaire. Based on the evaluations in the pilot test, the NiA-Q was adjusted into a final version. The estimated time to complete the NiA-Q was 30–45 min, depending on how many network members were named.

##### NiA-Q: Self-report

The newly developed NiA-Q starts by asking participants which life areas were important for them at this point in their life, because they wanted to learn or change something on a certain life area. The life areas were largely based on the life domains used in the Manchester Short Assessment of Quality of Life (Priebe et al., 1999). The life areas that participants could choose from were Physical functioning, Independent functioning, Psychological functioning, Housing, School/Work, Leisure activities, and Social contacts & Relationships. For each chosen life area, participants then filled out the names of a maximum of five persons that either supported or hindered them in this area. Subsequently, there was room to fill out names of persons that played a supporting or hindering role in their life, but who were not directly related to one of the chosen life areas (for this study categorized as belonging to the life area Other). So, the maximum number of network members that could be reported was 40 (maximum of five network members for a maximum of eight life areas). Next, participants gave factual information per named network member, namely their relationship with the person, living distance, frequency of face-to-face contact, and frequency of non-face-to-face contact. Then, the perceived social support from each network member was assessed in three ways. First, participants rated to which extent each network member was supportive for their chosen life area, which they answered on a three-point Likert scale (1 = not supportive to 3 = very supportive). Subsequently, this question was repeated, but now asking to which extent each network member was perceived as hindering. So, a network member could be rated as both supportive and hindering for a life area. Lastly, social support per named network member was investigated in a more comprehensive manner using twelve statements assessing *Emotional support* (e.g., *I can trust this person*; *this person makes me feel valued*), *Practical support* (e.g., *I do fun activities with this person*; *this person offers practical help, for example in case of sickness, in the household or in finance*) and *Interpersonal distress* (e.g., *this person negatively criticizes me*; *I have a conflict with this person*), also measured on a three-point Likert scale (1 = not supportive to 3 = very supportive). The scores on the items belonging to *Interpersonal distress* were reverse scored, so higher scores on the social support items indicate more emotional and practical support and less interpersonal distress. After the questions on social support, participants named the persons to whom they think they are supportive to themselves and were given room to explain why.

In the second part of the questionnaire, the focus was on their satisfaction and wishes with respect to their social network. First, the extent to which participants were content with their social network was rated on a five-point Likert scale (1 = not content at all to 5 = very content). This was followed by an open-ended question on what they wanted to change to become more content with their social network. Next, they indicated per named network member whether they wanted to involve them in learning new things or in helping to increase their wellbeing. Also, there was room to add new network members who they wanted to involve, but had not been named before in the questionnaire. In the subsequent open-ended question participants could describe in what manner they wanted to involve these network members. In the final question, the frequency of contacts with professional health care professionals of the last half year was filled out by participants.

##### NiA-Q: Proxy-Report

Similar to the self-report, the proxy-report version of the NiA-Q could be divided into two parts. In the first section, proxies were asked to answer the same questions about the social support and social network of the participants as in the self-report version. The only exception to this was that the proxy-version did not include the question to whom patients were supportive themselves. In the second part, proxies reported some demographic information (e.g., their age, sex, relationship to the participant and whether they were diagnosed themselves with autism) and their own perceived social support network, as the latter is associated to the proxy’s well-being (e.g., Barker et al., 2011; Smith et al., 2012), which in turn might influence their abilities to support the individual with an ASC. The questions followed largely the same format as the self-report version, but the proxy version was abbreviated. Proxies made a list of a maximum of ten network members who were supportive or hindering in the proxy’s care for the participant. For each named network member, they rated on a three-point Likert scale to which extent they were perceived as supporting and hindering, respectively. Next, they rated their contentment with their social contacts on a five-point Likert scale and were offered room to elaborate on their wishes with respect to their social contacts and how they liked to be supported by their network. In the current study, we only focused on the first part of the proxy version.

### Data Analysis

First, to check for potential confounders on descriptive measures between the complete sample of patients with an ASC and the group of patients with a corresponding proxy-report available, ANOVAs (for continuous variables) or Chi-square tests of independence/Fisher’s exact tests (for categorical variables) were performed.

Descriptive statistics were calculated to present the general characteristics of the social support network as reported by patients in the NiA-Q. Next, we tested the hypothesized three-factor structure of the twelve statements assessing *Emotional support*, *Practical support*, and *Interpersonal distress* of each network member. To test whether the hypothesized factor structure fitted the data, we ran a multilevel confirmatory factor analysis (CFA; for level 1 only, as this was the focused level of this study). For this analysis, the lavaan package (Rosseel, 2012) in R and the mcfa.input() function provided by Huang (2017) were used. We considered the chi-square test, the comparative fit index (CFI) that should be above 0.95 (Hooper et al., 2008), and the root mean square error approximation (RMSEA) that should be less than 0.06 (Hooper et al., 2008) of the hypothesized three-factor model of our study. In case the multilevel CFA would not confirm the three factor model, an exploratory factor analysis (EFA; maximum likelihood with promax rotation) in SPSS (version 25) would be conducted. As participants filled out the social support items multiple times (i.e., for each network member separately), one set of the twelve social support items was randomly chosen per participant for this EFA by generating a random number. In this way, complexities of a nested data structure, due to the dependencies in the data, were avoided (see Reise et al., 2005). The EFA was repeated two times, each with a new random set of the social support items, to cross-validate the results of the first EFA. Factor scores were calculated by summing the scores on the items loading at or above 0.40 on each factor (Norris & Lecavalier, 2010).

In addition, to compare the self- and proxy-report of the NiA-Q, paired sample t-tests were performed. In case there was more than one proxy report available for a patient, one of the proxy reports was randomly chosen for the analyses (by generating a random number in SPSS), leading to 84 pairs of self- and proxy-reports. Significant paired sample t-tests would indicate differences in self- and proxy-report at the group level. To check for influential effects of extreme values (defined as z-score above 3.29 or below − 3.29), the paired samples t-tests were repeated with extreme values replaced with *M* ± 3*SD. However, as this did not change the results, only the results of the first paired sample t-tests are reported. In addition to the paired sample t-tests, Bland–Altman plots were used to inspect for systematic differences across the range of scores for number of network members, perceived support, perceived hindering and number of network members to involve during treatment. In these plots, the value of the difference of each self–proxy rating pair was plotted on the y-axis and the value of the mean for each self–proxy rating pair was plotted on the x-axis. A positive mean difference score indicates a self-proxy pair where the proxy-report value is higher than the self-report value. Following the recommendation of Bland and Altman (Bland & Altman, 1986), regression analyses by regressing the mean on the difference score were performed to test for systematic trends across the range of scores. A significant regression coefficient indicates the presence of a systematic difference across the range of scores.

Besides the frequentist statistical analyses, we also performed Bayesian analyses to further explore the strength of the evidence for the results of the self- and proxy comparisons. These analyses were conducted in JASP version 0.13.1 (JASP Team, 2020). The prior was kept at the default (i.e., 0.707). We report the Bayes Factor_10_ (BF_10_), which indicates the likelihood that the alternative hypothesis is true compared to the null hypothesis. A BF_10_ of < 1 means no evidence for the alternative hypothesis over the null hypothesis (i.e., no difference), 1–3 anecdotal, 3–10 substantial, 10–30 strong, 30–100 very strong, and > 100 extreme evidence for the alternative hypothesis (Wagenmakers et al., 2011).

Lastly, the answers to the open-ended question of the NiA-Q (i.e., wishes for change in social network) were coded and presented in percentages per coding category for self- and proxy-report separately. For the wishes for change, the categories for coding of van Asselt-Goverts et al. (2015) were taken as starting point, so the categories: *More frequent contact*, *Better contact*, *Expanded network*, *Improved social skills* and *Other wishes*. Subsequently, we examined the answers within the category *Other wishes* whether additional, meaningful categories could be identified. Decisions with respect to coding and categorization were discussed among the researchers. In case more than one code could be applied to an answer, it received all applicable codes. Chi-square tests of independence or Fisher’s exact tests (in cases of < 5 expected counts per cell), percentage agreement and Krippendorff’s alpha (Hayes & Krippendorff, 2007) were used to compare the self- and proxy-report for the open-ended question per category (0 = category absent; 1 = category present). For these analyses, only the self-report of data patients who had a corresponding proxy-report were used.

## Results

### Sample Characteristics

In total, 292 adults participated in the study: 193 adults with an ASC and 99 proxies. Of the 128 of the 193 adults with an ASC that completed the Social Responsiveness Scale-Adult version (SRS-A) (Constantino, 2002), the majority scored above the cut-off of 54 (115 out of 128 adults). For a subset of 84 adults out of the total sample, at least one proxy completed the proxy-version of the NiA-Q. For 15 of these 84 adults, two proxy-reports were available. In this study, proxies were mother (37.3%, *n* = 37), partner (30.3%, *n* = 30), father (18.2%, *n* = 18), health care professional (7.1%, *n* = 7), other family member (6.1%, *n* = 6) or friend (1%, *n* = 1) of the patients. The complete sample of ASC and the subgroup with corresponding proxy-report available did not differ on demographic characteristics (see Table 1).

**Table 1:** Sample characteristics of patients with an Autism Spectrum Condition (ASC)

	Total ASC sample^a^ (*n* = 193)	Subsample with proxy-report (*n* = 84)	Statistics
Age (years)			
*M* (*SD*)	36.7 (12.0)	34.7 (12.3)	*F*(1, 275) = 1.66, *p* = .20
Range	18.1–71.3	18.1–65.9	
Sex (*n* (%))			χ^2^ (1) = 0.36, *p* = .55
Men	138 (71.5%)	63 (75.0%)	
Women	55 (28.5%)	21 (25.0%)	
SRS-A score (*M* (*SD*))^b^			
Total Range	89.1 (28.4) 25–174	89.7 (32.0) 25–174	*F*(1, 195) = 0.02, *p* = .89
Social awareness	25.0 (8.7)	25.0 (9.7)	
Communication	30.0 (11.1)	30.4 (12.3)	
Social motivation	19.6 (6.6)	19.8 (7.0)	
Rigiditity	14.6 (5.8)	14.5 (6.6)	
Living situation (*n* (%))			*p* = .59, Fisher’s exact
With parents	38 (19.7%)	23 (27.4%)	
Alone	53 (27.5%)	15 (17.9%)	
Together w. partner	65 (33.7%)	27 (32.1%)	
Together w. others	9 (4.7%)	4 (4.8%)	
Assisted living	7 (3.6%)	4 (4.8%)	
Clinical inpatient treatment	3 (1.6%)	2 (2.4%)	
Other	14 (7.3%)	6 (7.1%)	
Unknown	4 (2.1%)	3 (3.6%)	

^a^Adults with an ASC with and without corresponding Network in Action-Questionnaire proxy-report

^b^For the total sample: available for 128 of 193 participants. For the subsample with proxy-report: available for 69 of 84 participants

### General Characteristics

Patients reported on average 4.53 (*SD* = 1.95, median = 5) life areas that were important to them, with possible scores ranging from 0 to 8. They named on average 4.53 (*SD* = 3.78, median = 4) network members who were either hindering or supportive for all of their chosen life areas, where they could name a maximum of 5 persons per life area. The average level of experienced social support across the life areas was 2.44 (*SD* = 0.41, median = 2.5) and for perceived hindrance the average level was 1.55 (*SD* = 0.51, median = 1.49), where both variables could range from 1 to 3. Patients rated their level of satisfaction with their social network on average as 2.8 (*SD* = 1.08, median = 3), with possible scores from 1 to 5. They wanted to involve on average 3.13 (*SD* = 2.43, median = 3) network members in their treatment. Patients reported to be helpful themselves for on average 1.91 network members (*SD* = 1.79, median = 1), with a maximum possible number of 5 network members.

### Distance and Frequency of Contact

Most network members lived nearby; about a quarter (25.6%) lived between 0 to 5 km from the patients’ home. Roughly the same percentage lived between 5 to 30 km (24.4%) away. About an equal percentage of network members lived either in the same house as the patient (20.5%) or at least 30 km away (20.9%). A small number of network members lived in a different country (3.1%) or lived at unknown distance (5.5%).

About a fifth of the network members were seen on a daily basis by patients (21.7%). This was followed by face-to-face contact once per week (19.5%) and once per month (17.7%) with the network members. In 14.4% and 13.5% of the network members, patients saw them multiple times per week or per year, respectively. Lower frequencies yielded face-to-face contact once per year (8.9%) or unknown (4.4%).

With respect to contact other than face-to-face (e.g., via telephone or online), the most reported frequency was multiple times per week (18.6%), closely followed by daily contact (18.3%). Other reported frequencies were weekly (17.7%), monthly (15.3%), once a year (12.4%) and multiple times per year (9.1%). Frequency of non-face-to-face contact was reported as unknown in 8.6% of the cases.

### Factor Analysis

In the multilevel CFA, the covariance between the factor *Practical Support* and the factor *Emotional Support* was restricted to 0 as the covariance matrix appeared to be not positive definitive. The results of the multilevel CFA indicated that the hypothesized three-factor model did not fit the data well, χ^2^(52) = 435.316, *p* < 0.001, CFI = 0.795, RMSEA = 0.134. Following the analysis plan, an exploratory factor analysis was conducted. The number of factors was determined following the scree-test, which indicated two factors. This two-factor model explained 49.8% of the variance. The first factor consisted of all items related to positive social support, whereas the second factor was composed of items assessing interpersonal distress. One item (i.e., *this person has a bad influence on me (for example because of alcohol, drugs or criminality)*) had very low factor loadings on both factors. However, we decided to leave this item in the questionnaire, as we consider this item as measuring an important negative aspect of social influences, namely (potentially) risky behavior, which could have important consequences for treatment. See Table 2 for details on factor loadings. The two repeated EFAs resulted in the same two factor structure, although in one of the repeated EFAs the factor loadings of item 1, 10, and 11 were below 0.40 (between 0.31 and 0.38).

**Table 2:** Factor loadings in the exploratory factor analysis of the social support items of the Network in Action-Questionnaire (NiA-Q)

Social support item in NiA-Q	Factor 1	Factor 2
1. I can thrust this person	**0.59**	0.14
2. This person criticizes on me	− 0.20	**0.73**
3. I can talk to this person when I am upset, distressed or depressed	**0.83**	− .03
4. This person makes me feel valued	**0.58**	0.23
5. This person makes me feel unpleasant (for example, he/she bullies me)	− 0.03	**0.87**
6. This person understands my autism	**0.55**	0.20
7. I have a conflict/argument with this person	− 0.03	**0.73**
8. This person offers practical help (for example with illness, household, finances)	**0.63**	− 0.26
9. This person gives me information and advice	**0.67**	− 0.04
10. I do fun activities with this person	**0.43**	− 0.06
11. This person hinders me in what I want to do or achieve	0.16	**0.43**
12. This person has a bad influence on me (for example because of drugs, alcohol, crime)	0.21	− 0.17

Factor loadings larger than .40 are shown in bold

### Comparison of Ratings Between Self and Proxy

There was no difference in self- (*M* = 4.51, *SD* = 2.07) and proxy-report (*M* = 4.36, *SD* = 2.07) for the number of life areas that were important for the patient, *t*(83) = 0.53 *p* = 0.596, BF_10_ = 0.14. Proxies reported significantly more network members of patients (*M* = 5.61, *SD* = 3.34) than patients reported (*M* = 4.32, *SD* = 2.74), *t*(83) = − 2.99, *p* = 0.004*, d* = 0.42). The BF_10_ was 7.40, which indicated substantial difference in the two forms of report. However, patients’ ratings of social support (*M* = 2.45, *SD* = 0.41) did not significantly differ from the ratings by proxies (*M* = 2.47, *SD* = 0.39), *t*(75) = − 0.30, *p* = 0.764, BF_10_ = 0.13. Also, there was no significant difference in the perceived hindrance measured by self-report (*M* = 1.50, *SD* = 0.48) or proxy-report (*M* = 1.49, *SD* = 0.48), *t*(72) = 0.23, *p* = 0.821, BF_10_ = 0.13. Similarly, the self-report of the satisfaction with the social network (*M* = 2.84, *SD* = 1.08) did not significantly differ from the proxy-report (*M* = 2.69, *SD* = 0.89), *t*(76) = 1.27, *p* = 0.208, BF_10_ = 0.27. Furthermore, proxies wanted to involve more network members (*M* = 3.85, *SD* = 2.57) than patients (*M* = 3.11, *SD* = 2.25), but this difference did not reach significance, *t*(64) = − 1.97, *p* = 0.053*, d* = 0.31. The BF_10_ was 0.83, indicating no difference between the self- and proxy report. Lastly, the self-report and proxy-report of the two factor scores of the social support items were compared. The factor score for *Positive social support* of network members was significantly higher as reported by proxies (*M* = 2.38, *SD* = 0.33) than by patients (*M* = 2.3, *SD* = 0.33), *t*(65) = − 2.05, *p* = 0.044*, d* = 0.25. However, the BF_10_ was 0.96, which indicated no difference in the two forms of report. There was no difference in self- (*M* = 1.43, *SD* = 0.37) and proxy-report (*M* = 1.38, *SD* = 0.35) on the factor score concerning *Interpersonal distress* items, *t*(64) = − 1.05, *p* = 0.30, BF_10_ = 0.23.

**Fig. 1:**
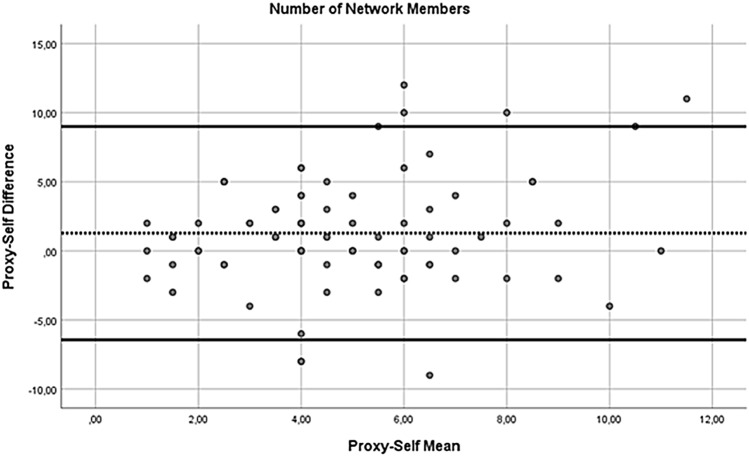
Bland–Altman plot of proxy-self differences for the number of network members. The dotted line represents the mean difference score and the solid lines show the limits of agreement (mean difference ± 1.96*SD; (Bland & Altman, 1986)). A positive mean difference score indicates a self-proxy pair where the proxy-report value is higher than the self-report value

**Fig. 2:**
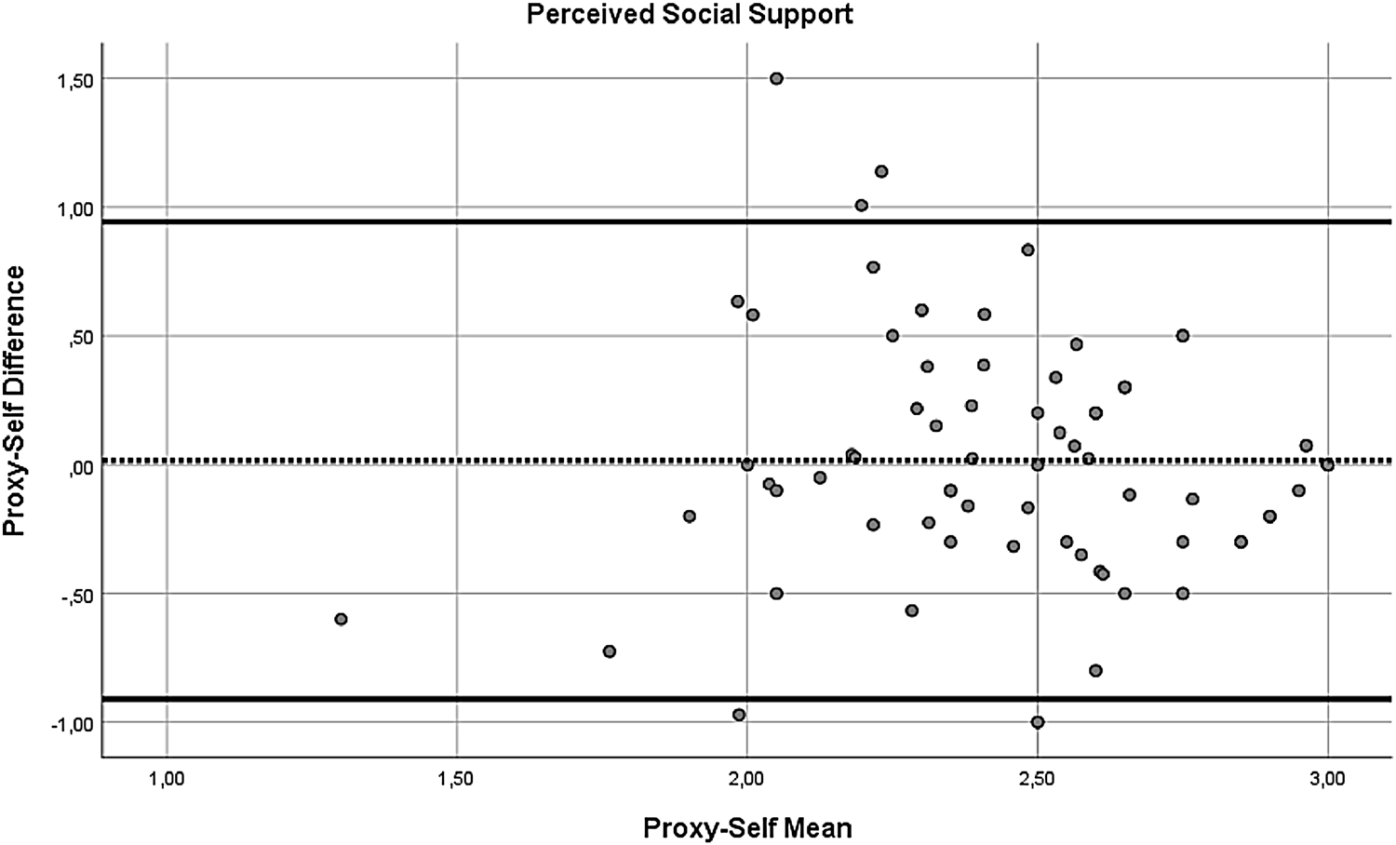
Bland–Altman plot of proxy-self differences for the perceived social support. The dotted line represents the mean difference score and the solid lines show the limits of agreement (mean difference ± 1.96*SD; (Bland & Altman, 1986)). A positive mean difference score indicates a self-proxy pair where the proxy-report value is higher than the self-report value

**Fig. 3:**
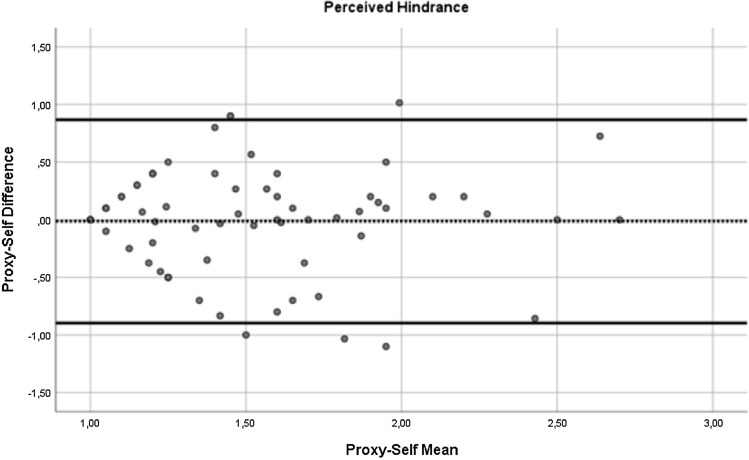
Bland–Altman plot of proxy-self differences for the perceived hindrance. The dotted line represents the mean difference score and the solid lines show the limits of agreement (mean difference ± 1.96*SD; (Bland & Altman, 1986)). A positive mean difference score indicates a self-proxy pair where the proxy-report value is higher than the self-report value

**Fig. 4:**
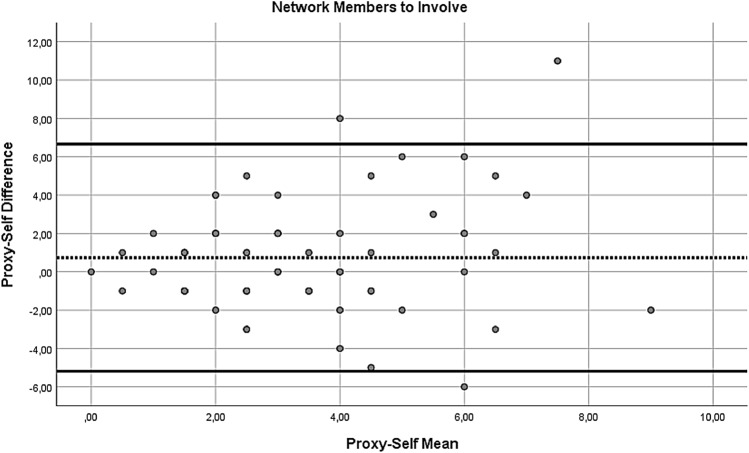
Bland–Altman plot of proxy-self differences for the number of network members to involve during treatment. The dotted line represents the mean difference score and the solid lines show the limits of agreement (mean difference ± 1.96*SD; (Bland & Altman, 1986)). A positive mean difference score indicates a self-proxy pair where the proxy-report value is higher than the self-report value

Next, we inspected Bland–Altman plots. For the number of network members (see Fig. 1), the mean self-proxy difference was 1.29 (*SD* = 3.94), with the most extreme differences being − 9 and + 12. The regression analysis of the mean on the difference score did not reach significance (*B* = 0.336, *t* = 1.838, *p* = 0.07), but it did indicate a non-significant trend of a larger discrepancy between the two informants with increasing number of reported network members. For the perceived social support (see Fig. 2), the mean self-proxy difference was 0.02 (*SD* = 0.47), with the most extreme differences being − 1 and + 1.5. The regression analysis indicated no significant relationship between the difference scores and means for perceived social support (*B* = − 0.088, *t* = − 0.526, *p* = 0.601). For the perceived hindrance (see Fig. 3), the mean self-proxy difference was − 0.01 (*SD* = 0.45), with the most extreme differences being − 1.1 and + 1.01. The regression analysis indicated no significant relationship between the difference scores and means for perceived hindrance (*B* = 0.000, *t* = − 0.004, *p* = 0.997). For the number of network members to involve (see Fig. 4), the mean self-proxy difference was 0.74 (*SD* = 3.02), with the most extreme differences being − 6 and 11. The regression analysis indicated no significant relationship between the difference scores and means for the number of network members to involve (*B* = 0.216, *t* = 1.079, *p* = 0.285).

### Exploratory Analyses

As the two factor scores of social support (i.e., *Positive social support* and *Interpersonal distress*) that emerged from the EFA were expected to correspond content-wise largely with two other variables within the NiA-Q which were both already captured in a single item (i.e., social support and perceived hindrance), we additionally took the opportunity to calculate Pearson correlations between these variables. In other words, we explored whether the more comprehensive measures of social support and interpersonal distress correlated with the corresponding single item measures for self- and proxy-report separately. The two self-report social support measures correlated strongly, *r*(163) = 0.58, *p* < 0.001, similar to the proxy-report measures, *r*(74) = 0.56, *p* < 0.001. The measures for perceived hindrance/interpersonal distress resulted in an equally strong correlation, both for the self-report *r*(161) = 0.63, *p* < 0.001, and the proxy-report, *r*(73) = 0.64, *p* < 0.001.

In addition, we explored whether the chosen life areas converged in the self- and proxy-report. As seen in Table 3, Krippendorff's alpha had very low values, indicating low agreement between patients and proxies.

**Table 3:** Selected life areas (in %) by patients with an Autism Spectrum Condition (ASC) and their proxies

Life areas	Total ASC sample^a^ (*n* = 193)	Subsample with proxy-report (*n* = 84)	Proxies (*n* = 84)	Krippendorff’s alpha^b^
Physical functioning	63.2	59.5	53.6	.11
Independent functioning	56.5	57.1	60.7	.39
Psychological functioning	86.5	83.3	77.4	-.09
Housing	33.2	31	36.9	.55
School/Work	66.3	76.2	60.7	.21
Leisure activities	51.8	51.2	44	.19
Social contacts and relationships	74.1	71.4	71.4	.07
Other	21.8	21.4	28.6	.18

^a^Adults with an ASC with and without corresponding Network in Action-Questionnaire proxy-report

^b^The statistics concern only the comparison between patients with corresponding proxy-report (*n* = 84) and proxies, so not the total sample of patients (*n* = 193)

### Wishes Regarding the Social Network

Based on the expressed wishes of patients and proxies regarding the social network of patients, we added four categories to the codes used in van Asselt-Goverts et al. (2015), namely *No wishes*, *Unable to formulate wishes*, *Wish for a romantic partner* and *Ambivalence*. This resulted in the original seven codes, namely: *More frequent contact* (with current network members; NB also including a minority of cases who wished less often contact), *Better contact* (qualitatively better contact with existing network members, such as increased understanding by network), *Expanded network* (wishing for new network members, such as more friends), *Improved social skills* (e.g., learn how to respond or increase self-esteem in social situations), and *Other wishes* (e.g., more spontaneous or longer lasting contact, taking more initiative for contact) plus adding the four new codes *No wishes* (content with the current network), *Unable to formulate wishes* (not knowing what in the network exactly they want to change), *Wish for a romantic partner*, and *Ambivalence* (wishing for more social contact, but realizing this would cost too much energy or would only lead to disappointment). The frequency of wishes regarding change of the social network of patients as reported by patients themselves and their proxies are presented in Table 4, as well as the statistics. Chi square tests or Fisher’s exact tests indicated that on the group level, there were almost no significant differences between self- and proxy-report on what kind of wishes they expressed. The only exception was that the distribution of having a wish for a romantic partner differed for patients and proxies. Patients reported more frequently a wish within the category *Improved social skills* than proxies, but this difference did not reach significance.

**Table 4:** Wishes regarding the social network (in %^a^) of the patient as reported by the total sample of patients with an Autism Spectrum Condition (ASC), ASC plus proxy-report and proxies

Category	ASC (*n* = 162)^b^	ASC with proxy-report (*n* = 62)	Proxies (*n* = 62)	χ^2^ (*df*) or Fisher’s exact^c^	Krippendorff’s alpha^c^	Percentage agreement^c^
More frequent contact	16.7	16.1	4.8	*p* = 1, Fisher’s exact	− .11	79.0
Better contact	34.6	32.3	27.4	χ^2^ (1) = 0.087, *p* = .77	− .03	56.5
Expanded network	43.2	43.5	51.6	χ^2^ (1) = 1.12, *p* = .29	.13	56.5
Improved social skills	25.3	21.0	8.1	*p* = .06, Fisher’s exact	.23	80.6
Wish for a romantic partner	7.4	6.5	6.5	*p* = .02, Fisher’s exact	.47	93.5
Ambivalence	10.5	12.9	9.7	*p* = .17, Fisher’s exact	.20	83.9
No wishes	11.1	12.9	11.3	*p* = 1, Fisher’s exact	.02	79.0
Unable to formulate wishes	3.1	1.6	6.5	*p* = 1, Fisher’s exact	− .04	91.9
Other wishes	7.4	9.7	11.3	*p* = 1, Fisher’s exact	− .11	79.0

^a^Percentages per group can exceed 100%, as wishes could be coded as belonging to more than one category

^b^Adults with an ASC with and without corresponding Network in Action-Questionnaire proxy-report

^c^The statistics concern only the comparison between patients with corresponding proxy-report (*n* = 62) and proxies, so not the total sample of patients (*n* = 162)

However, when manually inspecting the convergence between proxy-patient pairs on the individual level, 56.5% of the pairs did not have any overlap in coding of their expressed wish regarding the patient’s social network. If the pairs in which either the patient or the proxy had been unable to formulate a wish were left out, 52.6% of the pairs did not have any overlap in coding of their expressed wish. Krippendorff’s alpha and percentage agreement per category are presented in Table 4. Especially Krippendorff’s alpha shows very low agreement between the two raters. An example of such a difference can be seen in the following, where the patient (a 23 year old female) writes about ambivalence towards social contact, and the proxy (her mother) about her wish for her daughter to expand her network:

Patient’s wish: *Sometimes I would wish that I would like to have many persons around me. But I just don’t like that. I do not need many groups of people around me, but now I often feel alone. But when being together with others, I also often feel lonely. I do not really know what my wishes are with respect to this. Maybe I would want to feel less uncomfortable with social contact, for example when someone comes close to me or unexpectedly touches me. Now I suffer from this, except for the few people that are very close to me (family, friend).*

The proxy’s wish: *I would like her to have more friends of her own age to whom she could go to or have fun with.*

However, even if there was some overlap in coding between the answers of patients and proxies, their wishes could still vary considerably. This is demonstrated in the following example, of a 31 year old female with an ASC and her mother as her proxy. They both expressed a wish to expand the patient’s network, but the patient explicitly mentions the emotional connection that she desires in these new friendships, whereas the proxy’s wish only focusses more on increasing the number of contacts without referring to the quality of these new contacts.

Patient’s wish: *I would want to have more really good friends, with whom I could talk about my feelings and who have time to see me. Also, I would like to have a new, nice relationship*.

The proxy’s wish: *More people around her. Bigger network with for example neighbors, colleagues or people from a sport’s club.*

## Discussion

This study described a newly developed questionnaire for use in clinical practice, the NiA-Q, that facilitates the identification of possibilities for involving and strengthening the social support network during treatment. In addition, the results of the first use of the NiA-Q in adults with an ASC who registered for treatment at a mental health institution are presented. The analyses showed that instead of the expected three-factor structure on the items of social support a two-factor structure did fit the data. Exploration of the self-report of adults with an ASC showed that frequencies of face-to-face or other types of contact appeared to differ considerably per network member, as well as living distance between patient and network member. Comparison of the self- and proxy-report indicated that proxies reported on average more network members than patients reported themselves. However, for the other characteristics of the social support network no meaningful differences between the two forms of report were found. Most frequently reported wishes by patients and proxies for patient’s network were to expand the network with new network members and to have qualitatively better contact with existing network members. Nevertheless, in more than half of the patient-proxy pairs there was not any overlap in coding of their expressed wish regarding the patient’s social network.

The 12 social support items in the NiA-Q appeared to be best structured into two factors, namely *Positive support* and *Interpersonal distress*. This was in contrast to the factor structure that was aimed for during the construction of the questionnaire, consisting of the factors *Emotional support*, *Practical support* and *Interpersonal distress*. These three factors were originally chosen because of their association with mental health outcomes (e.g., Beutel et al., 2017; Smith et al., 2012) plus for their possibilities for clinical implications, as increasing the sources or frequency of practical support would probably require different actions than increasing emotional support. However, the items that were beforehand divided into the factors *Emotional support* and *Practical support* correlated strongly and could not be distinguished from one another in this data. This suggests that the items in the NiA-Q are not able to capture the distinct factors of *Emotional* and *Practical support*. However, an alternative explanation of this finding could be that the provision of emotional and practical support frequently coincides in the network members that were considered as important by the adults with an ASC. Previous research has shown that individuals with an ASC have on average a smaller network than nonautistic individuals (e.g., van Asselt-Goverts et al., 2015), which could suggest that the possible sources of social support within the network are more limited and therefore the same network members more often provide several forms of support. This would point at the importance of facilitating support for network members themselves if needed, as close network members (e.g., parents) of individuals with an ASC often experience elevated levels of stress (e.g., Hayes & Watson, 2013). An alternative strategy, in which the NiA-Q could be a useful tool, is to expand the social support network of patients with an ASC during treatment, because increasing the number of network members who can provide social support might lead to better balance of social support throughout the network. Importantly, enlarging the network is also in accordance with the wishes of patients with an ASC themselves, as almost half of the adults with an ASC in this study expressed a wish for expanding their social network.

In this study, the adults with an ASC seeking specialized mental health support reported to have on average four to five important network members, or at least one network member per important life area. This is in line with a study in a group of people with a serious mental illness (primarily diagnosis of schizophrenia, major depression or bipolar disorder), who named on average also around four important network members whom they could rely on for health-related issues (Pescosolido & Wright, 2004). A study in individuals with an ASC found a substantial higher number of network members (i.e., 11 network members) (van Asselt-Goverts et al., 2015). However, our sample is probably more comparable to the sample of Pescosolido and Wright (2004), as van Asselt-Goverts et al. (2015) included individuals with an ASC who received less specialized and less intensive forms of support than in our sample. Notably, the adults with an ASC (and their proxies) in the current study did not want to include all of their important network members during treatment, as the number of network members to involve was lower than the number of important network members. This highlights the importance of an instrument such as the NiA-Q to start a conversation between professional and patient about their needs and preferences on involvement of their social support network.

Although our analyses showed that on the group-level proxies reported more network members than patients reported themselves, closer inspection of the individual pairs via a Bland–Altman plot further clarified this finding. That is, the groups of proxies that reported less or more network members than patients seemed roughly balanced, so the statistical difference on group-level might rather be caused by a minority of proxies who reported far more network members than patients (e.g., a difference of 10 or more). Nevertheless, previous research found as well that proxies reported more network members than patients with a diagnosis of a serious mental illness reported themselves, which appeared largely attributional to a difference in perception of availability of persons at the center of the network (i.e., parent, sibling, child, or partner) (Pescosolido & Wright, 2004). Due to our data collection set-up, we could not reliably analyze whether patients and proxies reported the same persons as important network members, and therefore not test whether this finding applies to our sample as well. Keeping the goal of promoting involvement and strengthening the network during treatment in mind, it would be interesting to investigate the pairs in which proxies reported more network members than patients did themselves in more detail. Perhaps these additional network members reported by proxies but not by patients resemble currently underused or underperceived sources of social support within the network to whom patients could more often direct to for help. In other words, this information could be an interesting starting point in finding ways to strengthen the social support network during treatment. However, of utmost importance in this issue is to further investigate the perspective of the person with an ASC in each individual case, as he or she might have important reasons to not name this additional network member.

So, on the group-level, the perspective of proxies and patients agreed on most of the general aspects of the social support network in the current study. One could argue then that there is no strong need for an informant report on the social support network of an adult with an ASC, as it provides little new information compared to the self-report. However, there are two arguments why a proxy-report could still have additional value in clinical practice. First, incorporating a proxy-report can be helpful in light of promoting involvement of network members during treatment. That is, actively asking for their view on the current and potential sources of social support could be a method in itself to include network members during treatment and start a conversation between patient and network members on this topic, which alone might be a beneficial outcome of using the NiA-Q. Second, as seen in the results on patient’s and proxies wishes on the social network of patients, there can be relevant discrepancies between convergence on either the group-level or per individual patient-proxy pair. By comparing the two groups almost no meaningful differences in the frequencies per named theme appeared. However, by visually inspecting the individual patient-proxy pairs, only around half of the pairs had any overlap in which themes they mentioned in their wish. So again, the individual outcomes of the NiA-Q can start a conversation between patient and his or her important proxies on what their expectations and wishes are regarding the patient’s social network. During treatment, the professional can investigate together with the patient (and proxies) how there can be a balanced match between this person’s personal preferences versus what he or she receives as social support. For example, some patients expressed a form of ambiguity in their wish regarding their social network: they long for social contact with friends or family, but at the same time this is also exhausting for them. A qualitative study in adults with an ASC (Robledo & Donnellan, 2008) identified several factors for successful supportive relationships, such as a shared vision of independence and understanding from supporters. These could be relevant themes to discuss during treatment, in order to strengthen the social support as experienced by the patient.

Some might argue that it would be more efficient to directly discuss social network wishes with patients and proxies without filling out the NiA-Q beforehand, as this seems the most meaningful difference between self- and proxy-report. However, we believe first filling out the NiA-Q with subsequent discussion between patient and proxies could lead to more valid answers than directly discussing this in person with both patient and proxies together. That is, filling out the NiA-Q provides respondents with time to reflect on their views and wishes. Also, for some patients and/or proxies, it might be more difficult to express their genuine views or wishes when the persons concerned are present.

Furthermore, we found that around a quarter of the adults with an ASC in this study wished for improved social skills, in order to be more content with their social network, which is in line with van Asselt-Goverts et al. (2015). This is of course a valid wish to have, if this is what these adults with an ASC feel they need to develop to reach a better quality of life and, therefore, these desired social skills could be addressed during treatment. However, one can also look at this from the double-empathy perspective (Milton, 2012), which emphasizes that not only individuals with an ASC need to adjust themselves towards the neurotypical world and its expectations, but also vice versa. Looking from this point of view, perhaps one of the topics that could require attention during treatment is helping proxies how they can adjust their interaction or support towards the preferences of the individual with an ASC, in order to diminish distress experienced by individuals with an ASC.

This study has some limitations that are important to keep in mind when interpreting the results. First, it should be highlighted that the conclusions of this study might not be valid for all adults with an ASC, as we looked at a specific sample: adults with an ASC and without an ID who were seeking treatment at a specialized mental health institution, who often had co-occurring conditions and received previous, unsuccessful treatment. Future research should focus on the social support wishes and needs of adults with an ASC without co-occurring conditions. Second, due to the personalized structure of the NiA-Q, where (the number of) follow-up questions depended on previous answers on the questionnaire, there could have been differences between the exact format of the questionnaire per individual. For example, if patient A named six important network members and patient B reported four network members, patient A would subsequently receive six times a question on the supportiveness per network member (i.e., one for each network member), whereas patient B would receive four questions of this type. Related to this, proxies and patients could evaluate supportiveness on different network members, depending on which network members they named at the beginning of the questionnaire. So, this part of the results should probably be restricted to interpretations on the group level and primarily give an overall impression on self- and proxy-report on the social support network in adults with an ASC. Third, different types of proxies participated in the study, which could have influenced the degree of convergence between proxy- and self-report as the kind of relationship changes per type of proxy. This might specifically hold true for the few cases in which a professional was the proxy. Although professionals can play an important role in the network of individuals with an ASC (Robledo & Donnellan, 2008; van Asselt-Goverts et al., 2015), their relationship with the adult with ASC, and thereby their perspective on the details of the social network, could be of a different nature than in the case of an informal network member such as a partner or parent. However, the variety in proxy types is also a strength of this study, as patients were invited to ask the person whom they considered as a close and important network member, so we followed their preferences and did not use a standard proxy such as a mother. Fourth, a limitation of the development process of the NiA-Q is that we only included the perspectives of patients with an ASC and their proxies in the second development phase, and not also in the first phase. In addition, we did not investigate the feasibility and acceptability of the NiA-Q in this study, so these research questions should be addressed in a future study. Further, an avenue for future research could be to transform the NiA-Q into an interview format instead of a digital questionnaire, as some patients experienced their social network as a sensitive topic which they prefer to discuss directly with a professional. Also, the NiA-Q (or an interview equivalent) might be shortened by leaving out either the single-item measures of positive social support and perceived hindrance or the 12 items capturing the factors *Positive social support* and *Interpersonal distress*, as these corresponding constructs appeared to be strongly associated.

Concluding, the NiA-Q can facilitate professionals in the process of increasing involvement and strengthening of the social support network during treatment, based on the patients’ needs and wishes. Assisting in their preferences on their social network during treatment, could attribute to promoting quality of life in patients with an ASC.

## Footnotes

1 Throughout this manuscript, we use the term ‘patients’ to refer to individuals (with or without an ASC diagnosis) in a clinical setting, who are seeking treatment for mental health conditions to stress that we focus on this specific group and not on autistic adults in general.

2 The term ‘treatment’ in our manuscript does not refer to treatment of ASC itself, but rather to all types of interventions or combinations of multiple interventions that are used to increase mental health and well-being.

## References

[CR1] Alvarez‐Fernandez S, Brown HR, Zhao Y, Raithel JA, Bishop SL, Kern SB, Lord C, Petkova E, Martino AD (2017). Perceived social support in adults with autism spectrum disorder and attention-deficit/hyperactivity disorder. Autism Research.

[CR29] American Psychiatric Association. (2013). *Diagnostic and Statistical Manual of Mental Disorders (DSM-5®)*. American Psychiatric Pub.

[CR2] Barker ET, Hartley SL, Seltzer MM, Floyd FJ, Greenberg JS, Orsmond GI (2011). Trajectories of emotional well-being in mothers of adolescents and adults with autism. Developmental Psychology.

[CR3] Beutel ME, Brähler E, Wiltink J, Michal M, Klein EM, Jünger C, Wild PS, Münzel T, Blettner M, Lackner K, Nickels S, Tibubos AN (2017). Emotional and tangible social support in a German population-based sample: Development and validation of the Brief Social Support Scale (BS6). PLoS ONE.

[CR4] Bishop-Fitzpatrick L, Mazefsky CA, Eack SM (2018). The combined impact of social support and perceived stress on quality of life in adults with autism spectrum disorder and without intellectual disability. Autism.

[CR5] Bland JM, Altman DG (1986). Statistical methods for assessing agreement between two methods of clinical measurement. Lancet.

[CR6] Chronister JA, Johnson EK, Berven NL (2006). Measuring social support in rehabilitation. Disability and Rehabilitation.

[CR7] Constantino JN (2002). The social responsiveness scale—Adult version.

[CR8] Constantino JN, Gruber CP (2005). The social responsiveness scale manual.

[CR10] Deserno MK, Borsboom D, Begeer S, Geurts HM (2017). Multicausal systems ask for multicausal approaches: A network perspective on subjective well-being in individuals with autism spectrum disorder. Autism.

[CR11] Gariépy G, Honkaniemi H, Quesnel-Vallée A (2016). Social support and protection from depression: Systematic review of current findings in Western countries. British Journal of Psychiatry.

[CR13] Hanssen DJC, Rabeling-Keus IM, Lucassen PLBJ, Naarding P, van den Brink RHS, Comijs HC, Penninx BWJH, Oude Voshaar RC (2019). Measuring social support in psychiatric patients and controls: Validation and reliability of the shortened Close Persons Questionnaire. Journal of Psychiatric Research.

[CR14] Hayes AF, Krippendorff K (2007). Answering the call for a standard reliability measure for coding data. Communication Methods and Measures.

[CR15] Hayes SA, Watson SL (2013). The impact of parenting stress: A meta-analysis of studies comparing the experience of parenting stress in parents of children with and without autism spectrum disorder. Journal of Autism and Developmental Disorders.

[CR16] Hooper D, Coughlan J, Mullen MR (2008). Structural equation modelling: guidelines for determining model fit. Electronic Journal of Business Research Methods.

[CR102] Huang, F. L. (2017). Conducting multilevel confirmatory factor analysis using R. http://faculty.missouri.edu/huangf/data/mcfa/MCFA%20in%20R%20HUANG.pdf

[CR17] JASP Team. (2020). *JASP (Version 0.13.1)[Computer software]*.

[CR18] Kan, C. C. (Ed.), Geurts, H. M., van den Bosch, K., Forceville, E. J. M., van Manen, J., Schuurman, C. H., Sizoo, B. B., Stekelenburg, F., Veldboom, E., Verbeeck, W. J. C., Vrijmoed, D., & van Duin, D. (2013). *Multidisciplinaire richtlijn diagnostiek en behandeling van autismespectrumstoornissen bij volwassenen*. De Tijdstroom.

[CR19] Lever AG, Geurts HM (2018). Is older age associated with higher self- and other-rated ASD characteristics?. Journal of Autism and Developmental Disorders.

[CR20] Lyman DR, Braude L, George P, Dougherty RH, Daniels AS, Ghose SS, Delphin-Rittmon ME (2014). Consumer and family psychoeducation: Assessing the evidence. Psychiatric Services.

[CR21] Milton DEM (2012). On the ontological status of autism: The ‘double empathy problem’. Disability & Society.

[CR22] Müller E, Schuler A, Yates GB (2008). Social challenges and supports from the perspective of individuals with Asperger syndrome and other autism spectrum disabilities. Autism.

[CR12] National Institute for Health and Care Excellence. (2016, augustus 18). *1 Guidance | Autism spectrum disorder in adults: Diagnosis and management | Guidance | NICE*. NICE. https://www.nice.org.uk/guidance/cg142/chapter/1-Guidance 32186834

[CR23] Noens I, Steyaert J, De la Marche W, Scholte E (2012). Handleiding SRS-A—Screeningslijst voor autismespectrumstoornissen [SRS-A Social Responsiveness Scale, professional manual].

[CR24] Norris M, Lecavalier L (2010). Evaluating the use of exploratory factor analysis in developmental disability psychological research. Journal of Autism and Developmental Disorders.

[CR25] Osinga L, Botterblom S, Teunisse JP (2016). Netwerk in Actie (NiA): Het sociaal netwerk in de behandeling van mensen met autisme. Wetenschappelijk Tijdschrift Autisme.

[CR26] Pernice‐Duca F (2010). Family network support and mental health recovery. Journal of Marital and Family Therapy.

[CR27] Pescosolido BA, Wright ER (2004). The view from two worlds: The convergence of social network reports between mental health clients and their ties. Social Science & Medicine.

[CR28] Priebe S, Huxley P, Knight S, Evans S (1999). Application and results of the manchester short assessment of quality of life (Mansa). International Journal of Social Psychiatry.

[CR30] Reise SP, Ventura J, Nuechterlein KH, Kim KH (2005). An illustration of multilevel factor analysis. Journal of Personality Assessment.

[CR31] Renty JO, Roeyers H (2006). Quality of life in high-functioning adults with autism spectrum disorder: The predictive value of disability and support characteristics. Autism.

[CR32] Robledo JA, Donnellan AM (2008). Properties of supportive relationships from the perspective of academically successful individuals with autism. Intellectual and Developmental Disabilities.

[CR33] Rogers ES, Anthony W, Lyass A (2004). The nature and dimensions of social support among individuals with severe mental illnesses. Community Mental Health Journal.

[CR101] Rosseel Y (2012). lavaan: An R package for structural equation modeling and more Version 0.5-12 (BETA). Journal of Statistical Software.

[CR34] Sandercock RK, Lamarche EM, Klinger MR, Klinger LG (2020). Assessing the convergence of self-report and informant measures for adults with autism spectrum disorder. Autism.

[CR35] Santini ZI, Koyanagi A, Tyrovolas S, Mason C, Haro JM (2015). The association between social relationships and depression: A systematic review. Journal of Affective Disorders.

[CR36] Siette J, Gulea C, Priebe S (2015). Assessing social networks in patients with psychotic disorders: A systematic review of instruments. PLoS ONE.

[CR37] Smith LE, Greenberg JS, Seltzer MM (2012). Social support and well-being at mid-life among mothers of adolescents and adults with autism spectrum disorders. Journal of Autism and Developmental Disorders.

[CR38] Stansfeld S, Marmot M (1992). Deriving a survey measure of social support: The reliability and validity of the close persons questionnaire. Social Science & Medicine.

[CR39] Stein CH, Rappaport J, Seidman E (1995). Assessing the social networks of people with psychiatric disability from multiple perspectives. Community Mental Health Journal.

[CR100] Thomas D (2006). A general inductive approach for analyzing qualitative evaluation data. American Journal of Evaluation.

[CR40] Tracy EM, Whittaker JK (1990). The Social Network Map: Assessing social support in clinical practice. Families in Society: THe Journal of Contemporary Social Services.

[CR41] van Asselt-Goverts AE, Embregts PJCM, Hendriks AHC, Wegman KM, Teunisse JP (2015). Do social networks differ? Comparison of the social networks of people with intellectual disabilities, people with autism spectrum disorders and other people living in the community. Journal of Autism and Developmental Disorders.

[CR42] Wagenmakers EJ, Wetzels R, Borsboom D, van der Maas HL (2011). Why psychologists must change the way they analyze their data: The case of Psi: Comment on Bem (2011). Journal of Personality and Social Psychology.

[CR43] Zimet GD, Dahlem NW, Zimet SG, Farley GK (1988). The multidimensional scale of perceived social support. Journal of Personality Assessment.

